# Supplementary material for: Reassessing associations between white matter and behaviour with multimodal microstructural imaging
Source: Cortex. 2021 Dec;145:187–200. doi: 10.1016/j.cortex.2021.08.017 (PMC8940642; doi:10.1016/j.cortex.2021.08.017)
Supplement: Multimedia component 1 [file mmc1.docx]

# Supplementary Results


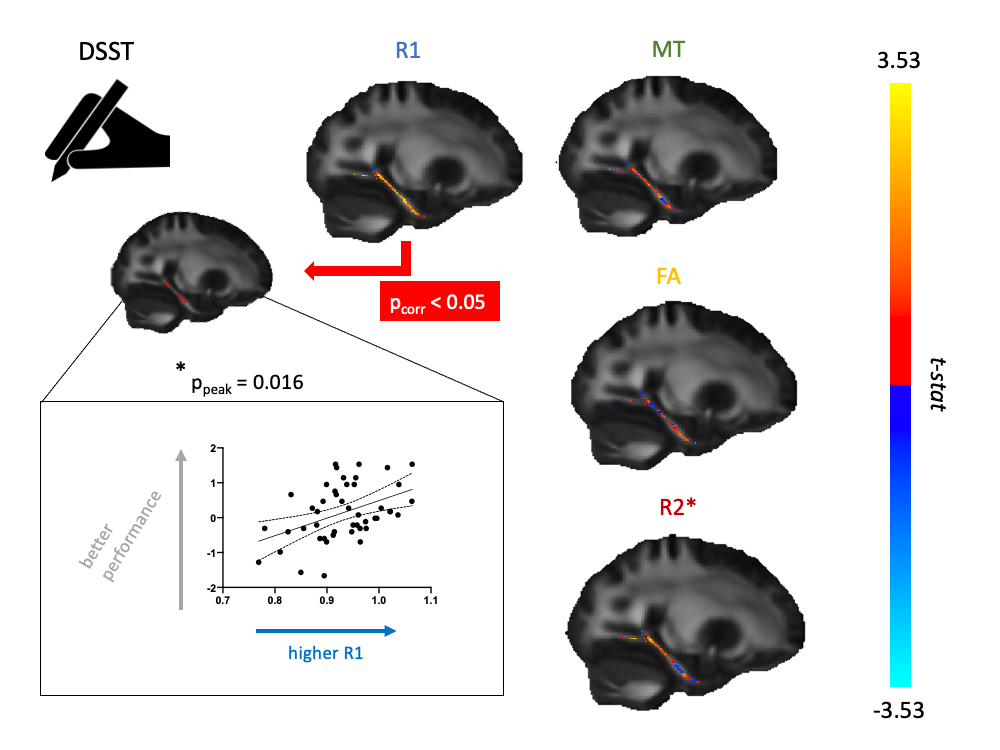


**Figure S1.** **Correlation between DSST performance and cingulum microstructure, reported as univariate results.** For each modality, unthresholded t-statistics are visualized according the colour bar (right). For R1 only, a cluster of voxels survived the threshold of p<0.05. Average R1 values within that cluster are shown against performance score in the scatterplot (with line of best fit and 95% confidence bands), which is presented for visualisation and is not used for statistical inference.


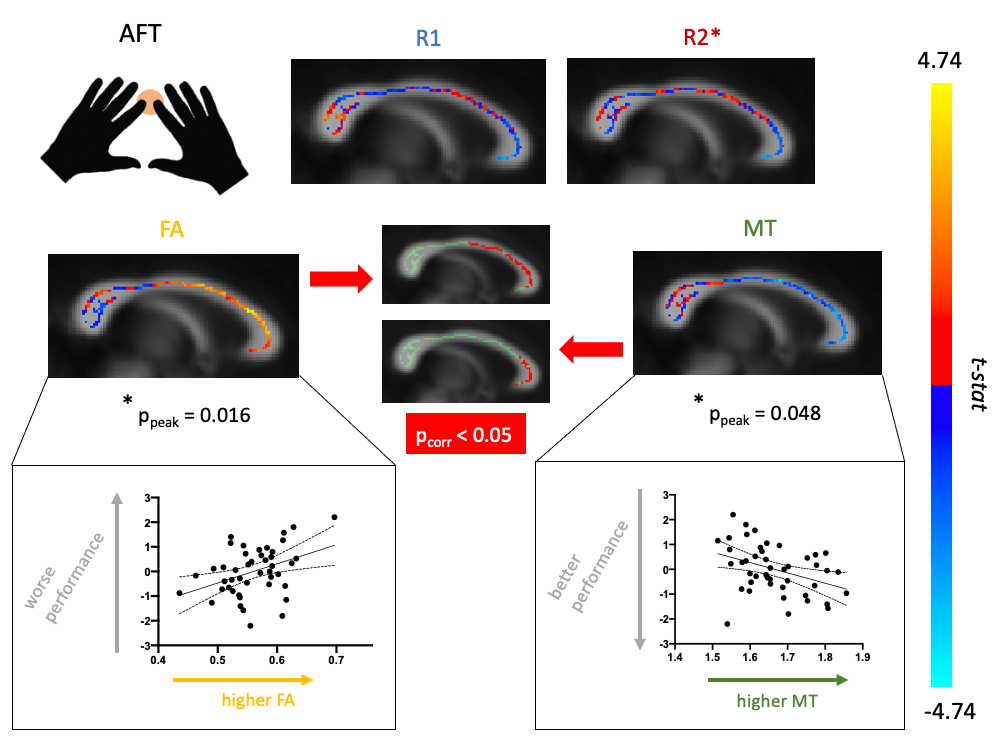


**Figure S2.** **Correlation between AFT and callosal microstructure, reported as univariate results.** For each modality, unthresholded t-statistics are visualized according the colour bar (right). For FA and MT, clusters of voxels survived the threshold of p<0.05. Average FA/MT values within that cluster are shown against performance score in the scatterplots (with line of best fit and 95% confidence bands), which are presented for visualisation and are not used for statistical inference.


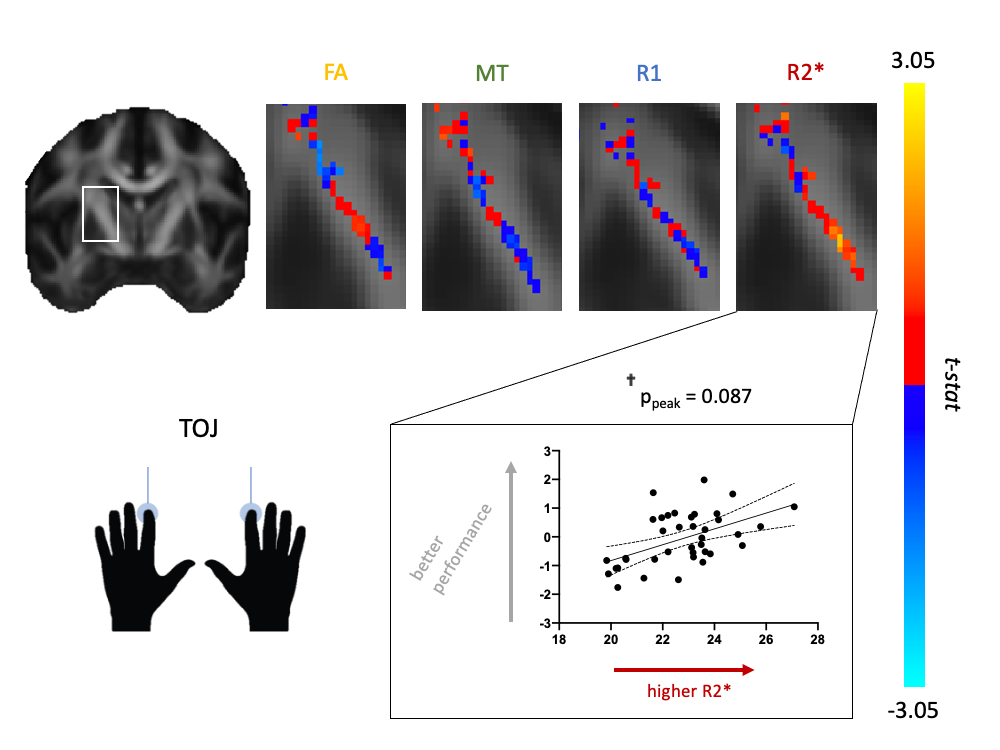


**Figure S3.** **Correlation between TOJ performance and CST microstructure, reported as univariate results.** For each modality, unthresholded t-statistics are visualized according to the colour bar (right). For R2* only, a cluster of voxels reached p=0.087. Average R2* values within that cluster are shown against performance score in the scatterplot (with line of best fit and 95% confidence bands), which is presented for visualisation and is not used for statistical inference.
